# Dissociation of amyloid biomarkers in PET and CSF in Alzheimer’s disease: a case report

**DOI:** 10.1186/s12883-015-0410-5

**Published:** 2015-08-26

**Authors:** Matthias L. Schroeter, Solveig Tiepolt, Anke Marschhauser, Angelika Thöne-Otto, Karl-Titus Hoffmann, Henryk Barthel, Hellmuth Obrig, Osama Sabri

**Affiliations:** Max Planck Institute for Human Cognitive and Brain Sciences, Stephanstraße 1A & Clinic for Cognitive Neurology, University of Leipzig, Liebigstr. 16 04103, Leipzig, Germany; Clinic for Cognitive Neurology, University of Leipzig, Leipzig, Germany; Leipzig Research Center for Civilization Diseases, University of Leipzig, Leipzig, Germany; German Consortium for Frontotemporal Lobar Degeneration, Ulm, Germany; Department of Nuclear Medicine, University of Leipzig, Leipzig, Germany; Department of Neuroradiology, University of Leipzig, Leipzig, Germany

**Keywords:** Alzheimer’s disease, Amyloid, CSF, MRI, PET

## Abstract

**Background:**

Recently, biomarkers have been suggested to be incorporated into diagnostic criteria for Alzheimer’s disease (AD). Regarding disease-specific brain amyloid-beta deposition these comprise low amyloid-beta 1–42 in cerebrospinal fluid (CSF) and positive positron emission tomography (PET) amyloid imaging, while neuronal degeneration is evidenced by high total and phosphorylated tau levels in CSF (t-/p-tau), regional hypometabolism ([^18^F]fluorodeoxyglucose PET, FDG-PET) and characteristic atrophy-patterns (magnetic resonance imaging, MRI).

**Case presentation:**

Here we present a case of clinically and biomarker supported AD (CSF t-/p-tau, MRI, FDG-PET) in a 59-year-old Caucasian man in whom indicators of amyloid-beta deposition dissociated between CSF parameters and the respective PET imaging.

**Conclusions:**

Such cases highlight the necessity to better understand potential dissociations between PET and CSF data for amyloid-beta biomarkers, because they are currently considered interchangeably valid with regard to *in-vivo* evidence for AD pathology. This is more important since amyloid deposition markers can be considered a very first prognostic indicator of imminent AD, prior to neurodegenerative biomarkers and cognitive symptoms. The case illustrates the need for further longitudinal data on potential dissociations of AD biomarkers to devise recommendations for their better prognostic and diagnostic interpretation in the future.

## Background

Recently, biomarkers have been suggested to be incorporated into diagnostic criteria for Alzheimer’s disease (AD) [1, 2]. Evidence for amyloid-beta protein deposition in the brain is provided by low amyloid-beta 1–42 in cerebrospinal fluid (CSF) and positive positron emission tomography (PET) amyloid imaging. For downstream neuronal injury and degeneration elevated total tau (t-tau) and phosphorylated tau (p-tau) levels in CSF, decreased [^18^F]fluorodeoxyglucose uptake on PET (FDG-PET) in temporoparietal and posterior cingulate cortices, and atrophy in temporal and medial parietal cortices on structural magnetic resonance imaging (MRI) have been established to yield a high diagnostic precision [2]. Biomarkers for brain amyloid-beta deposition may be considered especially important since they provide very early *in-vivo* evidence of imminent Alzheimer’s pathology, beside proven AD autosomal dominant mutations in relevant genes such as presenilin and amyloid precursor protein.

Amyloid PET imaging detects amyloid-beta plaques with high accuracy as shown in several clinical trials [3]. Accordingly, three [^18^F]-labeled amyloid-beta-targeting radiotracers have been approved by the Food and Drug Administration and the European Medicines Agency. However, introduction of amyloid-beta PET imaging into everyday clinical practice requires validation also in the diagnostic process of single patients to allow personalized evidence-based medicine. Beyond large scale trials, potential inconsistencies between different disease-specific biomarkers in a single patient, as shown here, require guidelines for clinical practice.

## Case presentation

### Clinical assessment

A 59-year-old Caucasian man presented with progressive cognitive decline in the memory domain since 18 months. Memory impairments were relevant in everyday life and during his work in a garage. Furthermore, he reported problems in dual task management. Cognition was investigated in several domains (for references see [4–6]): the test battery of the Consortium to Establish a Registry for Alzheimer’s Disease (CERAD plus, including Mini Mental State test, MMST) for global cognitive functioning, the Bayer Activities of Daily Living (B-ADL) scale for daily life functioning, subtests of the Testbatterie zur Aufmerksamkeitsprüfung (TAP) for alertness and divided attention, the Behavioral Assessment of the Dysexecutive Syndrome (BADS) for executive functions, and subtests of the Wechsler Memory Scale (WMS-IV logical memory and visual reproduction) to assess memory functions. Behavioral impairments were investigated with the Neuropsychiatric Inventory (NPI), as well as the Apathy Evaluation Scale (AES).

Comprehensive neuropsychological testing revealed deficits mainly in learning and memory (CERAD wordlist learning total/recall z = −2.09/−1.99; figures copying/recall z = 0.81/−3.65; WMS-IV logical memory immediate recall/delayed recall/recognition percentile rank < 1/<1/2, visual reproduction immediate recall/delayed recall/recognition percentile rank = 5/1/2), in attention (CERAD trail making test A/B z = −2.46/−1,40; TAP tonic/phasic alertness percentile rank = 10/4) and executive functions (CERAD phonematic/semantic fluency z = 0.18/−1.17; BADS action programme, key search, zoo profile score = 4/4,3/4,0/4). Executive functions were mainly impaired due to deficits in encoding complex instructions, while planning and error monitoring seemed fairly normal. The MMSE score was 26/30, which indicates mild cognitive impairment (MCI). Note that the B-ADL scale excluded severe impairments in activities of daily living as assessed by the patient’s spouse, although the patient had to quit work due to severe memory deficits, which interfered with his highly responsible job. The NPI identified only minor, unspecific behavioral symptoms in the domains depression, apathy, and irritability/lability ruling out behavioral variant frontotemporal dementia (score 1 for each domain, total score 3) [7]. This was confirmed by scores below cut-off in the AES. Progression of cognitive dysfunction was based on subjective evaluation by the patient and his relatives (wife and daughter). A neuropsychological screening three months before our comprehensive examination showed comparable results.

### Biomarker assessment

As recommended by the National Institute on Aging-Alzheimer’s Association [2], clinical testing was supplemented by biomarker investigations for brain amyloid-beta protein deposition (amyloid PET imaging using [^18^F]florbetaben as an amyloid imaging biomarker, amyloid-beta 1–42 levels in CSF), and for neuronal degeneration/injury (t-/p-tau in CSF, structural MRI and FDG-PET).

**Fig. 1:**
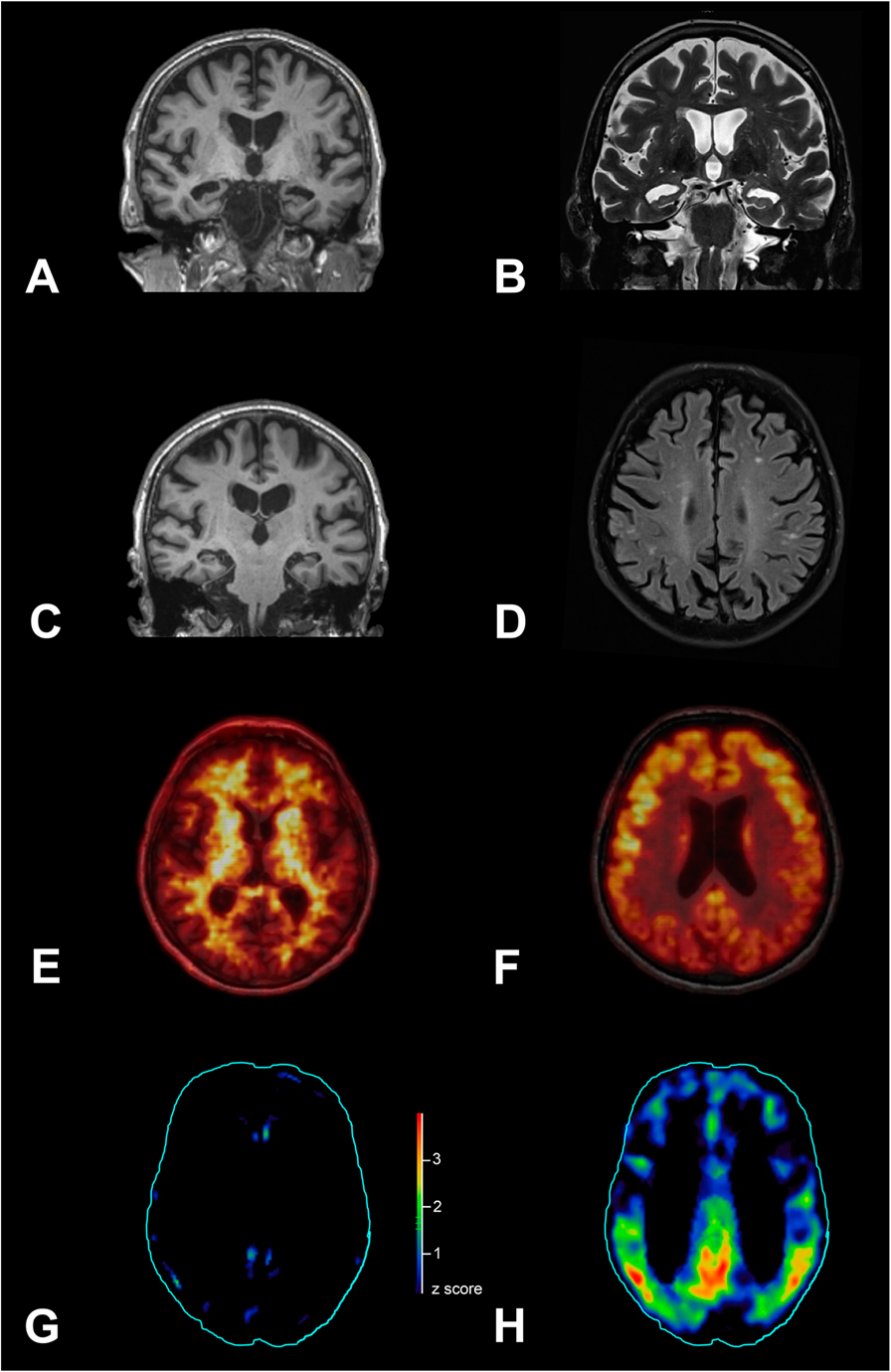
Imaging biomarkers for Alzheimer’s disease. Atrophy was evident in magnetic resonance imaging (MRI) in temporal/hippocampal regions in T1 (**a**) and T2 (**b**) sequence, and in parietal cortex in T1 (**c**). Single white matter lesions were detected in fast fluid-attenuated inversion recovery (FLAIR) images (**d**). Amyloid positron emission tomography (PET) with [^18^F]florbetaben did not show specific binding in the neocortical gray matter (**e**), although hypometabolism was detected in bilateral parietotemporal and posterior cingluate cortices with [^18^F]fluorodeoxyglucose-PET (**f**). Finally, we evaluated statistical deviation of tracer uptake from a normal control group including 94 (amyloid) or 25 (glucose) normal subjects with the Hermes Brass Software. Z score map of the [^18^F]florbetaben PET (**g**) did not show relevant (z score > 2.5) cortical tracer uptake increase, whereas the z score map of [^18^F]fluorodeoxyglucose-PET (**h**) showed relevant (z score > 2.5) tracer uptake reduction in bilateral parietotemporal regions and posterior cingulate cortices

As illustrated in Fig. 1, structural MR imaging revealed hippocampal (Scheltens score 4) and parietal atrophy. MRI revealed multiple small non-confluent white matter lesions indicating only mild small vessel disease, but no other pathology. CSF biomarkers showed a typical AD pattern with decreased amyloid-beta 1–42 (345.3 pg/ml; reference value > 450), increased p-tau (78.7 pg/ml; reference value < 61), and slightly elevated t-tau (439.9 pg/ml; borderline value 300–450, pathological > 450). All other CSF-parameters were normal apart from a mildly increased total protein (547.7 mg/l; reference value 200–500). Surprisingly, amyloid PET with [^18^F]florbetaben did not show elevated tracer uptake throughout the whole gray matter (Fig. 1). The composite standard uptake value ratio - an established measure of the mean brain amyloid load [8, 9] - equalled 1.09 (threshold < 1.39) thus not suggesting an AD pathology. All other biomarkers supported AD including a highly AD-typical pattern for glucose hypometabolism (FDG-PET) in bilateral parietotemporal and posterior cingulate cortex as illustrated in Fig. 1 [10]. The apolipoprotein E status was E3/E3.

The comprehensive clinical and paraclinical examination confirmed the diagnosis of AD, supported by biomarkers (CSF, MRI, FDG-PET) [1, 2], although the investigations revealed a dissociation between PET and CSF data for brain amyloid-beta deposition. Other metabolic, traumatic and medical causes of cognitive symptoms were ruled out.

## Conclusions

Biomarkers indicating amyloid-beta deposition in the brain have been suggested to yield *in-vivo* evidence of Alzheimer’s pathology. Here, we show that biomarkers for amyloid-beta deposition can be dissociated between PET and CSF. Notably all clinical and further biomarkers (CSF t-tau and p-tau, MRI, FDG-PET) supported the diagnosis of AD. In line with our finding, a recent study [11] including amyloid-beta PET and CSF levels has shown a considerable dissociation between [^18^F]florbetapir PET and amyloid-beta 1–42 in CSF in 20 out of 374 subjects (5.3 %) ranging from normal to MCI and AD (103, 249, 22 subjects, respectively). The 13 participants showing a dissociation with *abnormal* [^18^F]florbetapir PET vs. *normal* CSF amyloid-beta 1–42 were normal control subjects or early MCI cases. The remaining seven participants with more substantial cognitive deficits and higher CSF t-tau (one AD, four late MCI, one early MCI, one normal) showed the reverse dissociation with *normal* [^18^F]florbetapir PET vs. *abnormal* CSF amyloid-beta 1–42. The latter pattern is confirmed in the here reported case of late MCI/early dementia due to AD and, though it may be rare in AD (one in 22 AD patients, accordingly approximately 4.5 % in the above mentioned study), it is of relevance to the individualized counselling of patients. With respect to Landau et al.’s [11] and our findings one might hypothesize specific dissociations in the course of AD with positive amyloid PET and normal CSF amyloid-beta 1–42 in early MCI and a reverse pattern with decreased CSF amyloid-beta 1–42 and normal amyloid PET in late MCI or clinically evident AD (dementia stage), although this assumption is based on a small number of cases warranting caution with regard to generalization.

Generally, Landau et al. [11] reported a robust negative correlation between both biomarkers for brain amyloid deposition as shown by [^18^F]florbetapir PET retention and CSF amyloid-beta 1–42 levels in their AD cohort. This correlation was more robust than correlations with t-tau or p-tau. Such a negative correlation was also shown in another study with [^11^C]Pittsburgh compound B (PIB) PET retention and levels of CSF amyloid-beta 1–42 [12]. The latter study additionally revealed a significant negative correlation between CSF amyloid-beta 1–42 levels and [^11^C]PIB PET retention in parietal (including the precuneus) and temporal cortices, the posterior cingulate, frontal and primary visual cortex. Interestingly, such a correlation was not established for t-tau or p-tau. Both effects in this study [12] were only observed when patients with AD (37) and its prestage MCI (21) were pooled, highlighting potential effects of the comparatively low sample size.

In conclusion, our case report shows that biomarkers for amyloid-beta deposition can be dissociated between PET and CSF in clinically and biomarker supported otherwise typical AD. This finding is of special relevance because amyloid biomarkers have been suggested to be incorporated into diagnostic criteria for AD [1, 2]. Understanding potential dissociations between PET and CSF data for amyloid-beta biomarkers is of particular relevance, because these biomarkers are currently considered to interchangeably yield very early *in-vivo* evidence of AD pathology prior to neurodegenerative biomarkers and cognitive symptoms [13]. Accordingly, further studies and longitudinal data are warranted to interpret such dissociated AD biomarker patterns and develop recommendations on how to cope with these discrepancies in diagnostic procedures in the future.

## Consent

Written informed consent for publication of this Case Report and any accompanying images was obtained from the patient. A copy of the written consent is available for review to the Editor of this journal.

## Abbreviations

AD: Alzheimer’s disease
CSF: Cerebrospinal fluid
FDG-PET: [^18^F]fluorodeoxyglucose positron emission tomography
MCI: Mild cognitive impairment
MRI: Magnetic resonance imaging
PET: Positron emission tomography
PIB: [^11^C]Pittsburgh compound B
p-tau: Phosphorylated tau
t-tau: Total tau
